# Activation of neurons in the insular cortex and lateral hypothalamus during food anticipatory period caused by food restriction in mice

**DOI:** 10.1186/s12576-023-00892-2

**Published:** 2023-12-08

**Authors:** Jihao Ma, Sakurako Yanase, Lisa Udagawa, Tomoyuki Kuwaki, Ikue Kusumoto-Yoshida

**Affiliations:** Department of Physiology, Graduate School of Medical and Dental Sciences, Kagoshima University, Kagoshima, 890-8544 Japan

**Keywords:** Insular cortex, Lateral hypothalamus, Orexin neuron, Food anticipatory activity, c-Fos expression, Restricted feeding

## Abstract

Mice fed a single meal daily at a fixed time display food anticipatory activity (FAA). It has been reported that the insular cortex (IC) plays an essential role in food anticipation, and lateral hypothalamus (LH) regulates the expression of FAA. However, how these areas contribute to FAA production is still unclear. Thus, we examined the temporal and spatial activation pattern of neurons in the IC and LH during the food anticipation period to determine their role in FAA establishment. We observed an increase of c-Fos-positive neurons in the IC and LH, including orexin neurons of male adult C57BL/6 mice. These neurons were gradually activated from the 1st day to 15th day of restricted feeding. The activation of these brain regions, however, peaked at a distinct point in the food restriction procedure. These results suggest that the IC and LH are differently involved in the neural network for FAA production.

## Background

Eating is an animal's motivational behavior of seeking, obtaining, and consuming food driven by the craving instinct [1]. The neural regulation of feeding behavior is a very complex process. First, the nervous system senses the energy state of the body, the appearance, smell, taste, and nutrients of food, and uses these information as food anticipatory clues [2–4]. This sensory information is transmitted and integrated by the highly complex feeding regulation neural network, which sends instructions to regulate feeding behavior [5–7]. Food anticipatory cues drive not only feeding behavior but also the body's expectancy of food. The expectation also motivates the body to seek out and consume food [8–10]. In humans, foraging behavior driven by food anticipation is manifested as an increase in food intake [11], which may lead to obesity, diabetes, and other metabolic diseases. Therefore, it is essential to understand the neuronal mechanism of food anticipation.

It has been reported that mice fed a single daily meal at intervals within the circadian range show increased locomotor activity in the precedent feeding period, considered food anticipatory activity (FAA) [12]. However, the mechanism of FAA production is still unclear. The insular cortex (IC) is a higher-order sensory cortex that integrates multiple modalities, such as taste and visceral information [13–15]. IC also has been suggested to play an essential role in food anticipation and control of taste-guided, reward-directed choices and actions [16–18]. The previous study showed that the lesions of male Wistar rats' bilateral anterior agranular IC by electrolysis or ibotenic acid significantly increased the FAA, suggesting that the anterior agranular IC contributes to the network of brain regions involved in FAA [19]. Yet it remains unclear whether the neurons in the IC are activated during FAA and play roles in FAA production. Several subregions (anterior (AI), middle (MI) and posterior (PI) regions) in the IC are known to have different connections with different brain regions and functions [20–25]**.** Human brain imaging research shows that the AI exhibits high responsiveness to both anticipated food intake and actual food intake, indicating a significant association between AI and the processes involved in both the anticipation and actual intake of food [16]. Sensory perception of food-related stimuli (including visual, olfactory, and taste) leads to increased activation of the AI and dorsal parts of the MI in human subject [18]. Therefore, to clarify whether the AI, MI, and PI also play different roles in FAA production, we examined the activation patterns of neurons in the three subregions of IC during the food anticipatory period.

The lateral hypothalamus (LH) is related to the control of primitive motivational behaviors, including feeding and energy homeostasis [26–30]. Previous research has shown that c-Fos expression in LH increases during FAA in mice [12]. Orexin neurons in LH enhance appetite and food consumption, play an important role in feeding behavior regulation [29, 31], increase spatial memory for food [32], and are required for the robust expression of FAA in mice during the meal anticipation period [33]. Although it has been explored that neurons in LH, including orexin neurons, participate in FAA [12, 33], the temporal patterns of activation of these neurons in LH during the development of FAA, and interrelationships of the temporal pattern between IC and LH remain to be confirmed.

To corroborate the establishment of FAA, the current study examined the effects of daily scheduled food restriction for 4 h on c-Fos expression during the food anticipatory period in the bilateral IC and LH. Furthermore, the development of c-Fos expression was observed on the day-1, 8, and 15 of restricted feeding to investigate how IC and LH neurons are activated during food restriction protocol, and possible time-related changes that may lead to the identification of the brain network responsible for the formation of FAA.

## Methods

### Animals

Male C57BL/6 mice (*n* = 60) were weight (20–30 g) and 8–12 weeks old at the beginning of the experiment. The animals were singly housed in laboratory mouse cages (17.2 cm × 10 cm × 11 cm) in the experiment room seven days before the start of the food restriction to adapt to the experimental environment, under standard laboratory conditions 12:12 h light to dark (12:12 LD, lights on at 7:00 defined as Zeitgeber Time 0 (ZT0), and off at 19:00 as ZT12) cycle, with a constant room temperature (23 °C) and humidity (51%). All the mice received standard murine chow (CE-2, CLEA JAPAN, INC) and water available ad libitum.

### Feeding schedules and behavioral recording

**Fig. 1:**
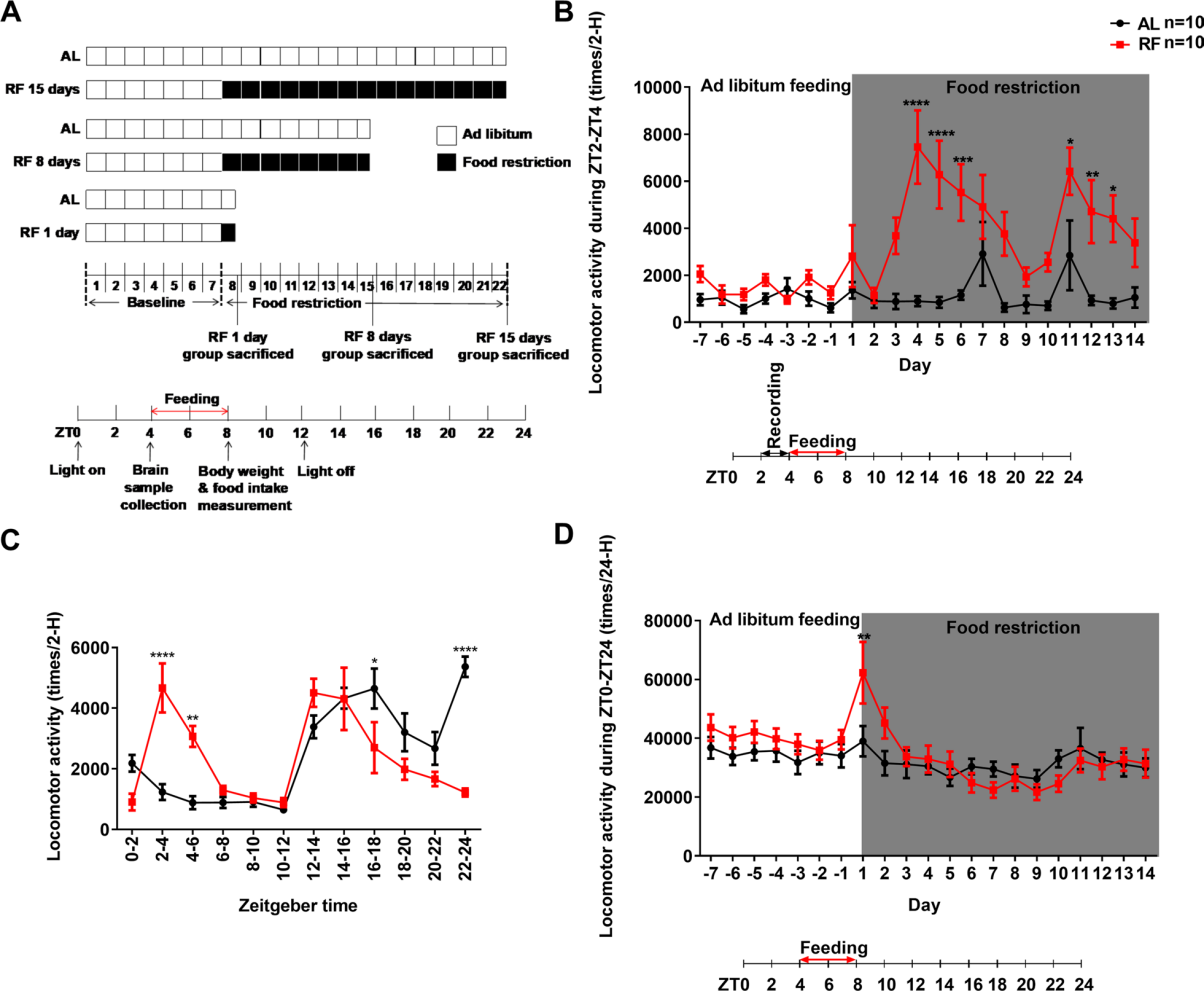
Experimental schedule and locomotor activity of mice in response to 15 days of restricted feeding. **A** Experimental schedule. After one week of baseline recording, mice in the ad libitum (AL) groups received free access to food throughout the study. In contrast, mice in the restricted feeding (RF) (1 day, 8 days, 15 days) groups were allowed access to food between ZT4-ZT8 during the days (for 1 day, 8 days, and 15 days, respectively). **B** Time course of the food anticipation activity (FAA) in response to 15 days of restricted feeding. **C** Mean locomotor activity of mice from day 4 to day 14 of restricted feeding. The mean (± SEM) locomotor activity for ten subjects is shown on the y-axis. The x-axis represents Zeitgeber time. **D** Time course of the 24-H locomotor activity in response to 15 days of food restriction. A similar trend in the time course, except for the first day of the food restriction, was observed in AL and RF groups (**D**). The mean (± SEM) locomotor activity during ZT2-ZT4 (**B**) or ZT0-ZT24 (**D**) for ten subjects is shown on the y-axis. The x-axis represents experimental days (**B** and **D**). *P < 0.05; **P < 0.01; ***P < 0.001; ****P < 0.0001 difference between AL mice and RF mice according to Bonferroni’s multiple comparison test

Mice were individually housed in the same cage mentioned above, with bedding on the bottom and an infrared motion sensor (AMN 1111, Panasonic Co., Osaka, Japan) on the top of the lid. Mice were assigned into two groups with their body weight counterbalanced, and the restricted feeding group (RF) received restricted access to food for 4 h (from ZT4 to ZT8). The ad libitum feeding group (AL) received free access to food throughout the study. Mice in the RF and AL groups were further divided into 6 groups (RF 1 day, RF 8 days, RF 15 days group, and their corresponding control AL groups). During the first 7 days of the experiment, all the animals received ad libitum feeding to record baseline data. After the week, mice in RF groups were restricted to accessing food for 4 h (from ZT4-ZT8) for 1 day, 8 days, and 15 days, respectively, while mice in AL groups received free access to food throughout the study (1 day, 8 days,15 days, respectively) (Fig. 1A). Each day at ZT4, newly measured chows were given to both AL and RF groups. At ZT8, newly calculated chows were prepared and provided for the AL groups but not for RF groups. The mice's body weight and food intake amount were measured at the timing of ZT8. All mice in RF 1 day, 8 days,15 days groups and their control AL groups were sacrificed at ZT4 on the last day of the RF, respectively (note: RF groups with no food delivery) (Fig. 1A).

The behavioral activity of the mice was continually collected in 6-min bins throughout the study by the infrared motion sensor connected to a computer and expressed as the total counts in bins per 2 h or 24 h.

### Immunohistochemistry

Mice were deeply anesthetized with urethane (1.8 g/kg, intraperitoneal injection), and perfused transcardially with 25 ml of phosphate buffer (PB, 0.1 M, pH 7.4) followed by 25 ml of 4% paraformaldehyde (PFA) solution in PB. The whole head was detached and fixed in 4% PFA solution at 4 °C overnight, and the brain was removed and fixed in 4% PFA solution at 4 °C overnight for 1 day. Slicing the brain to a series of 40 μm using a vibratome (SuperMicroSlicer Zero1; DOSAKA EM, Kyoto, Japan), every 4th section was used for immunostaining. For double labeling of c-Fos and orexin, tissues were immersed in a blocking solution (1% normal horse serum and 0.3% Triton-X in 0.01 M PBS) for 30 min at room temperature. Primary antibodies were dissolved in blocking solution, and the conditions were as follows: guinea pig anti-c-Fos antibody (226004, Synaptic Systems, RRID AB_2619946) at 1/1000 at room temperature for 1 h; goat anti-orexin antibody (SC-8070, Santa Cruz Biotechnology, RRID AB_653610) at 1/200 at room temperature for 1 h. The sections were washed with PBS 3 times, each for 10 min. And tissues were incubated with a secondary antibody diluted in a blocking solution. The secondary antibodies were as follows: anti-Guinea pig IgG-biotin (706-065-148, Jackson ImmunoResearch Laboratories, INC, RRID AB_2340451) at 1/250 at room temperature for 1.5 h; anti-goat IgG-CF568 (20106, Biotium, RRID AB_10559672) at 1/200 at room temperature for 1.5 h. The sections were washed with PBS 3 times and each for 10 min. Streptavidin Alexa488 (S11223, Invitrogen) was used to visualize c-Fos at 1/200 in PBS at room temperature for 1.5 h. Then, it was washed with PBS 3 times, each time for 10 min.

All the sections were mounted on microscope slides (PRO-02, Matsunami, Osaka, Japan) and covered with a cover glass (C024601, Matsunami) after using mounting reagent Mowiol (Sigma Aldrich) with DAPI. Finally, sections were observed under a fluorescence microscope (BZ-X700, Keyence, Osaka, Japan).

### Area setting of histological analysis

To identify the number of c-Fos positive neurons in the IC, we counted neurons by positioning a counting container bilaterally over a region of interest. For the AI, we placed a 500 × 500 µm square box on Sections 2.58–1.37 mm rostral to bregma; For the MI, we put a 500 × 500 µm box on Sections 1.37–0.16 mm rostral to bregma; For the PI, we set a 500 × 500 µm box on Sections 0.16 mm (rostral to bregma) to 1.06 mm (caudal to bregma) based on brain atlas [34]. To identify the number of c-Fos positive neurons and orexin neurons in the LH, we placed a 1200 × 400 µm (width × height) rectangular box on sections from 0.94 mm to 2.18 mm caudal to bregma. We selected three slices on the left and right from the brain slices of mice to observe the brain areas of interest. The number of positive neurons on each side was calculated separately, and the average value of the number of positive neurons on each side was used for statistics. Cell counting was done with Adobe Photoshop CC software (Adobe Systems Inc., San Jose, CA, USA) and expressed as the number of cells per unit area of 0.25 mm^2^ in AI, MI, and PI and per unit area of 0.48 mm^2^ in LH.

### Statistical analysis

Data were presented as Mean ± SEM. GraphPad Prism software (version 7, La Jolla, California, USA) was used for statistical analysis**.** Data on the mice's locomotor activity, food intake, and body weight were analyzed using the repeated measures two-way ANOVA followed by Bonferroni’s multiple comparison test. Histology data were analyzed using an unpaired *t*-test with Welch’s correction, one-way ANOVA followed by Bonferroni’s multiple comparison test, or Pearson's correlation coefficient. The results were considered significant at *P* < 0.05.

## Results

In rodents, a standard daily restricted feeding paradigm is often utilized, alternating between feeding 2–5 h and fasting 19–22 h daily for 7 days to 3 weeks [12, 19, 33, 35–37], to study the mechanism of FAA production. It has been reported that rodents express FAA within 3–14 days under the scheduled restricted feeding paradigm [38]. Some brain structures began significantly increasing c-Fos expression on the 8th day of palatable food entrainment [39]. Therefore, we selected day 15 (14 days of RF and 20-h fasting) and day 8 (7 days of RF and 20-h fasting) of RF for locomotor activity and c-Fos histochemical studies. In addition, to determine whether 20-h fasting can produce FAA and its effect on the expression of c-Fos in IC and LH neurons, we also observed the effect of 1-day RF (20-h fasting) on locomotor activity and the expression of c-Fos in IC and LH neurons as a control for day 8 or day 15 of RF.

### The development of food anticipatory activity

In previous studies, it has been reported that FAA begins 2–3 h before food delivery [12]. To confirm whether we could also observe a similar increase in locomotor activity in the pre-mealtime in our experimental setting, we investigated the effects of daily scheduled restricted feeding on the locomotor activity of mice. Figure 1B shows the development of FAA of mice in 15 days of RF schedule. RF-mice's locomotor activity transition during ZT2-ZT4 (2 h before the feeding) differed significantly from AL-mice (feeding-effect: F(1, 18) = 17.27, *P* = 0.0006; day-effect: F(20, 360) = 6.284, *P* < 0.0001; interaction: F(20, 360) = 4.124, *P* < 0.0001, repeated measures two-way ANOVA). RF-mice showed significantly higher locomotor activity after three days of RF (*P* < 0.0001, Bonferroni’s test). It then remained higher than AL-mice during the rest of the RF schedule. The results suggest that the appearance of FAA is gradual and requires repeated, predictable stimulation of scheduled feeding restrictions (at least three times). However, the strength of FAA fluctuated rather than remained constant during the daily scheduled restricted feeding (Fig. 1B), which is similar to previous research reports [12, 33]. Figure 1C shows the mean locomotor activity of mice from day 4 to day 14 of restricted feeding, i.e., after significant enhancement of FAA was observed in RF-group. There was a significant difference in the interaction of feeding and time between AL- and RF-group (feeding-effect: F(1, 18) = 0.2882, *P* = 0.5979; time-effect: F(11, 198) = 18.09, *P* < 0.0001; interaction: F(11, 198) = 11.98, *P* < 0.0001, repeated measures two-way ANOVA, Fig. 1C). The locomotor activity of RF-mice was significantly higher during ZT2-ZT4 (*P* < 0.0001, Bonferroni’s test).

In addition, in the daily total locomotor activity during ZT0-ZT24, there was a similar decreasing tendency between these two groups except for the first day of food restriction (feeding-effect: F(1, 18) = 0.3942, *P* = 0.5380; day-effect: F(20, 360) = 7.975, *P* < 0.0001; interaction: F(20, 360) = 2.860, *P* < 0.0001, repeated measures two-way ANOVA; *P* = 0.0015, Bonferroni’s test (day1), Fig. 1D). The results showed that 15 days of food restriction to ZT4-ZT8 did not affect the movement capability of mice but could cause significant alterations in daily locomotor activity patterns (an enhancement of locomotor activity during ZT2-ZT4).

### The changes in food intake and body weight during daily scheduled RF

**Fig. 2:**
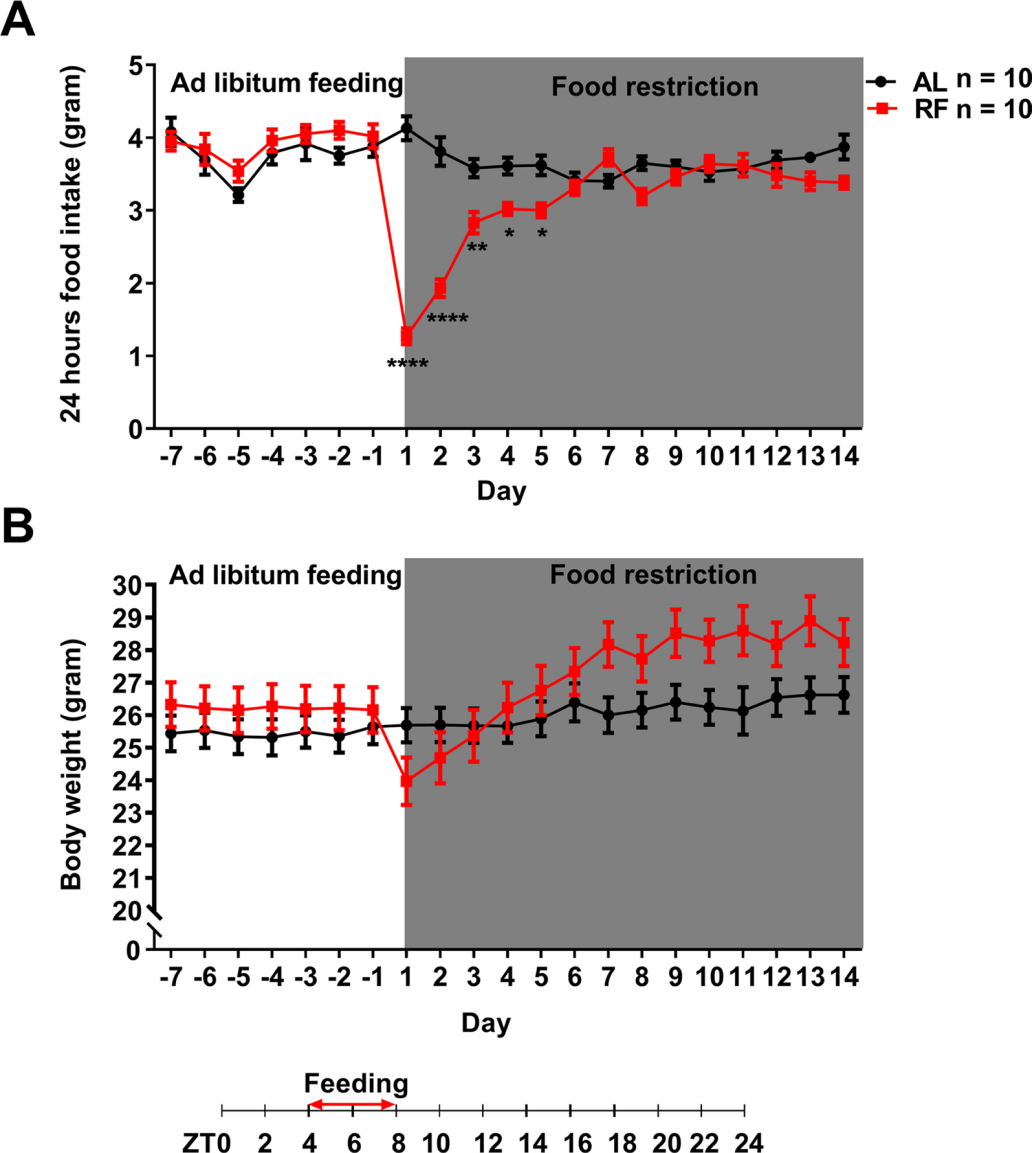
Changes in daily food intake and body weight during15 days restricted feeding. Time course of the daily food intake (**A**) and changes in daily body weight (**B**) in response to 15 days of food restriction in mice. The mean (± SEM) daily food intake (**A**) or body weight (**B**) for ten subjects is shown on the y-axis. The x-axis represents experimental days. *P < 0.05; **P < 0.01; ****P < 0.0001 difference between AL mice and RF mice according to Bonferroni’s multiple comparison test

We speculated that the RF mice might reduce their daily food intake due to time-limited access to food. Therefore, we examined the effect of daily scheduled RF on the overall daily food intake (Fig. 2A). The food intake between AL-group and RF-group showed a significant difference (feeding-effect: F(1, 18) = 16.26, *P* = 0.0008; day-effect: F(20, 360) = 14.91, *P* < 0.0001; interaction: F(20, 360) = 18.80, *P* < 0.0001, repeated measures two-way ANOVA). Compared with AL-mice, the food intake of the RF-mice decreased significantly on the first day of food restriction (*P* < 0.0001, Bonferroni's test). However, it recovered gradually with the number of days and reached a similar level to AL-mice after five days of food restriction (*P* > 0.05). Thus, limited food access reduced food intake only at the early phase of the protocol.

The RF mice were also concerned about losing their body weight because of the food restriction. Compared with the AL group, the change in body weight of the RF group was significantly different in the interaction of feeding and day (feeding-effect: F(1, 18) = 1.285, *P* = 0.2719; day-effect: F(20, 360) = 44.20, *P* < 0.0001; interaction: F(20, 360) = 16.82, *P* < 0.0001, repeated measures two-way ANOVA, Fig. 2B). On the first day of feeding restriction, the body weight of the RF-mice decreased significantly by 8.6% (*P* < 0.0001, Bonferroni’s test), then recovered and increased gradually with the number of the days (*P* > 0.05 on the 4th day of feeding restriction), then kept at a high level (increased by 5.8% to 10.3%) after six days of feeding restriction (*P* < 0.001). According to the findings, the RF between ZT4 and ZT8 only caused a drop in the mice's daily body weight during the early stages of the food restriction but an increase during the latter stages. The maximum body weight loss of the RF-mice in our study was 8.6% of their original body weight, within the range (loss of less than 20%) of the requirements of experimental animal ethics [40, 41].

### The activation of neurons in the IC during the food anticipatory period

**Fig. 3:**
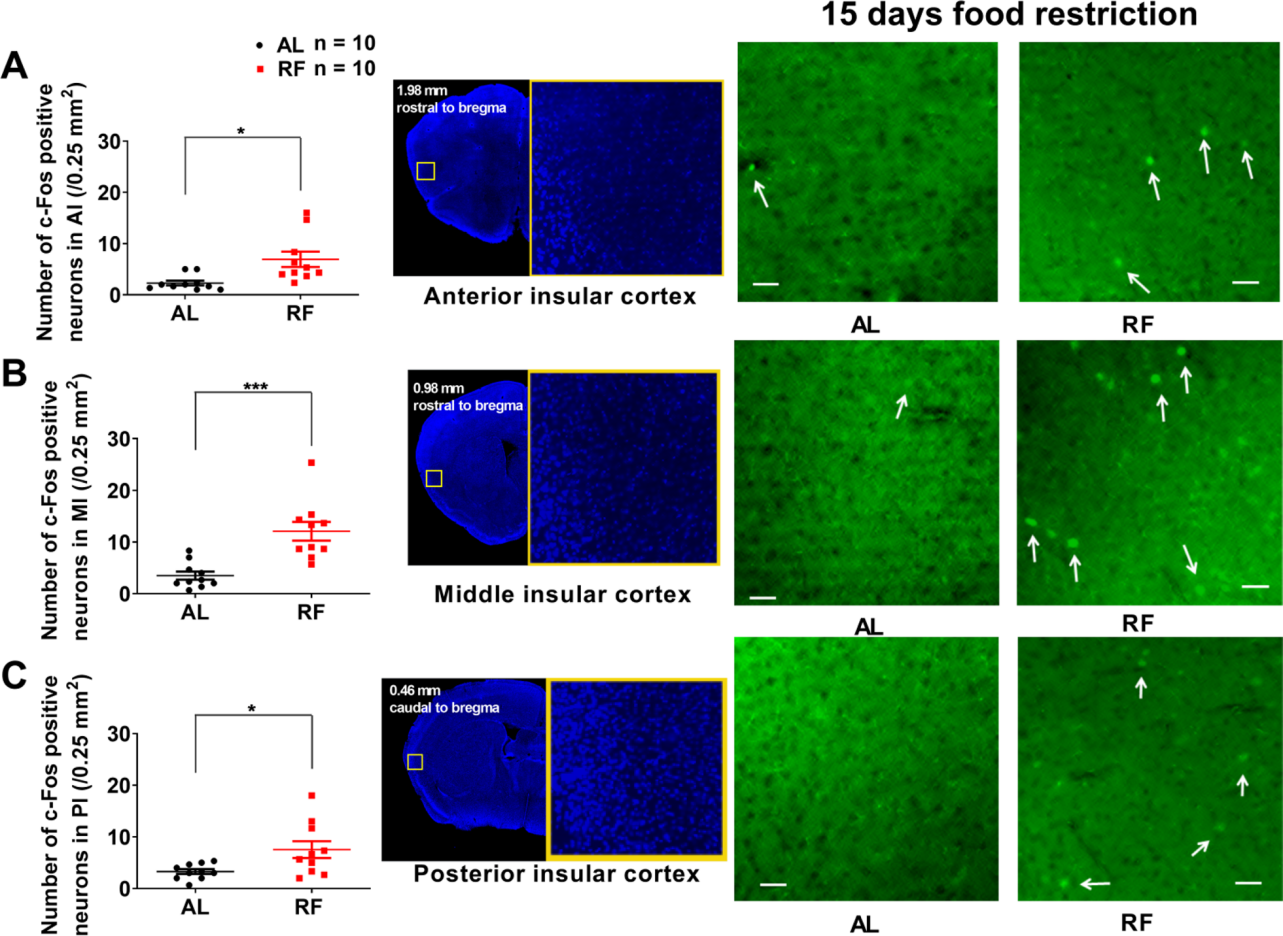
Increase in the number of c-Fos-positive neurons in IC of 15 days RF-mice. **A**, **B**, and **C** The number of c-Fos positive neurons counted within a square (500 × 500 μm) in the anterior (**A**), middle (**B**), and posterior (**C**) insular cortex in ad libitum feeding (AL) and 15 days of restricted feeding (RF). A significantly higher number of c-Fos was observed in RF than in AL. **A**–**C**, Left. The mean (± SEM) of c-Fos positive neurons for ten subjects are shown on the y-axis. *P < 0.05; ***P < 0.001 difference between AL mice and RF mice according to unpaired *t*-test with Welch’s correction. **A**–**C**, Middle. The tallied regions in AI, MI, and PI are marked with yellow squares in representative mouse brain sections counterstained with DAPI. **A**–**C**, Right. Photographs of representative sections showing c-Fos positive neurons in AI (**A**, Right), MI (**B**, Right), and PI (**C**, Right) from AL and RF mice. Arrows indicate representative c-Fos signals (**A**–**C**, Right). Green represents the c-Fos signal. The scale bar is 50 μm

We examined whether IC neurons were activated during FAA to investigate the neuronal involvement of IC in FAA production. We sacrificed all mice at ZT4 (during FAA) on the 15th day of RF (note: RF-group with no food delivery). We examined three subregions of IC separately. Compared with AL-mice, the number of c-Fos positive neurons of 15 days RF-mice increased significantly in AI (AL: 2.3 ± 0.5/0.25 mm^2^, RF: 6.9 ± 1.5/0.25 mm^2^, *n* = 10, t = 2.959, *P* = 0.0143), MI (AL: 3.5 ± 0.8/0.25 mm^2^, RF: 12.1 ± 1.8/0.25 mm^2^, *n* = 10, t = 4.351, *P* = 0.0009), and PI (AL: 3.3 ± 0.5/0.25 mm^2^, RF: 7.5 ± 1.6/0.25 mm^2^, *n* = 10, t = 2.495, *P* = 0.0317, unpaired *t*-test with Welch’s correction) (Fig. 3A–C). There was no significant difference in c-Fos expression between the bilateral IC's neurons of both the RF- and AL-mice (This paper only shows the experimental data of the left side IC as a representative. The data of the right-side IC are not shown unless specifically mentioned). The results indicate that the bilateral AI, MI, and PI neurons are activated during ZT2-ZT4 and may contribute to the network of brain regions involved in FAA [12].

### The activation of neurons in the LH, including orexin neurons, during the food anticipatory period

**Fig. 4:**
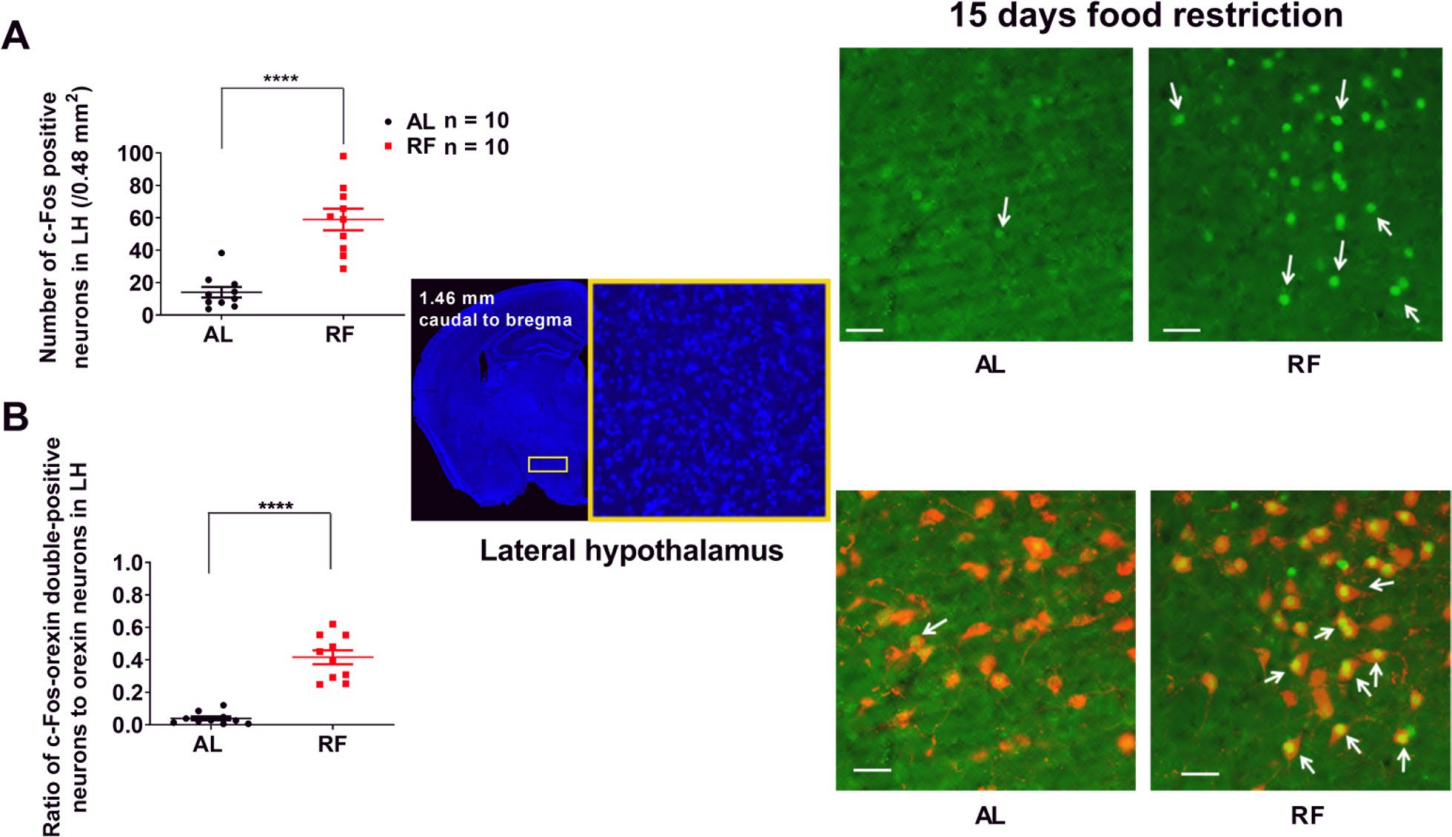
Increases in the number of c-Fos-positive and c-Fos-orexin-double-positive neurons in LH of 15 days RF-mice. **A** and **B** The number of c-Fos positive neurons (**A**) and the ratio of c-Fos-orexin double-positive neurons to the orexin neurons (**B**) counted within a rectangle (1200 × 400 μm) in the lateral hypothalamus (LH) in ad libitum feeding (AL) and 15 days of restricted feeding (RF). RF group showed significantly higher values. **A** and **B**, Left. The mean (± SEM) of c-Fos positive neurons (**A**, Left) and c-Fos-orexin double-positive neurons ratio in orexin neurons (**B**, Left) for ten subjects are shown on the y-axis. ****P < 0.0001 difference between AL mice and RF mice according to unpaired *t*-test with Welch’s correction. **A** and **B**, Middle. The tallied region in LH is marked with a yellow rectangle in the representative section of the mouse brain counterstained with DAPI. **A** and **B**, Right. Photographs of representative sections showing c-Fos positive neurons in LH (**A**, Right) and c-Fos positive orexin neurons in LH (B, Right) from AL and RF mice. Arrows indicate representative c-Fos signals (**A**, Right) and representative c-Fos-orexin signals (**B** Right). Green represents the c-Fos signal, and red represents the orexin signal. The scale bar is 50 μm

To confirm whether the neurons, including orexin neurons in LH, were also activated during FAA in our experimental setting, we examined the effect of daily scheduled RF on the number of c-Fos expressing neurons in LH during FAA. Compared with AL-mice, the number of c-Fos positive neurons in LH of 15 days RF-mice increased significantly (AL: 14.1 ± 3.3/0.48 mm^2^, RF: 58.9 ± 6.7/0.48 mm^2^, *n* = 10, t = 6.046, *P* < 0.0001, unpaired *t*-test with Welch’s correction, Fig. 4A). The same tendency was also observed in orexin-neurons (proportion of c-Fos positive orexin neurons in counted orexin neurons; AL: 0.040 ± 0.012, RF: 0.416 ± 0.043, *n* = 10, t = 8.435, *P* < 0.0001, Fig. 4B). There was no significant difference in c-Fos expression between the bilateral LH neurons or orexin neurons. The results indicate that the neurons, including orexin neurons, in the bilateral LH are activated during ZT2-ZT4 and may contribute to the network of brain regions involved in FAA.

### How does activation of neurons in the bilateral IC develop during food restriction protocol?

**Fig. 5:**
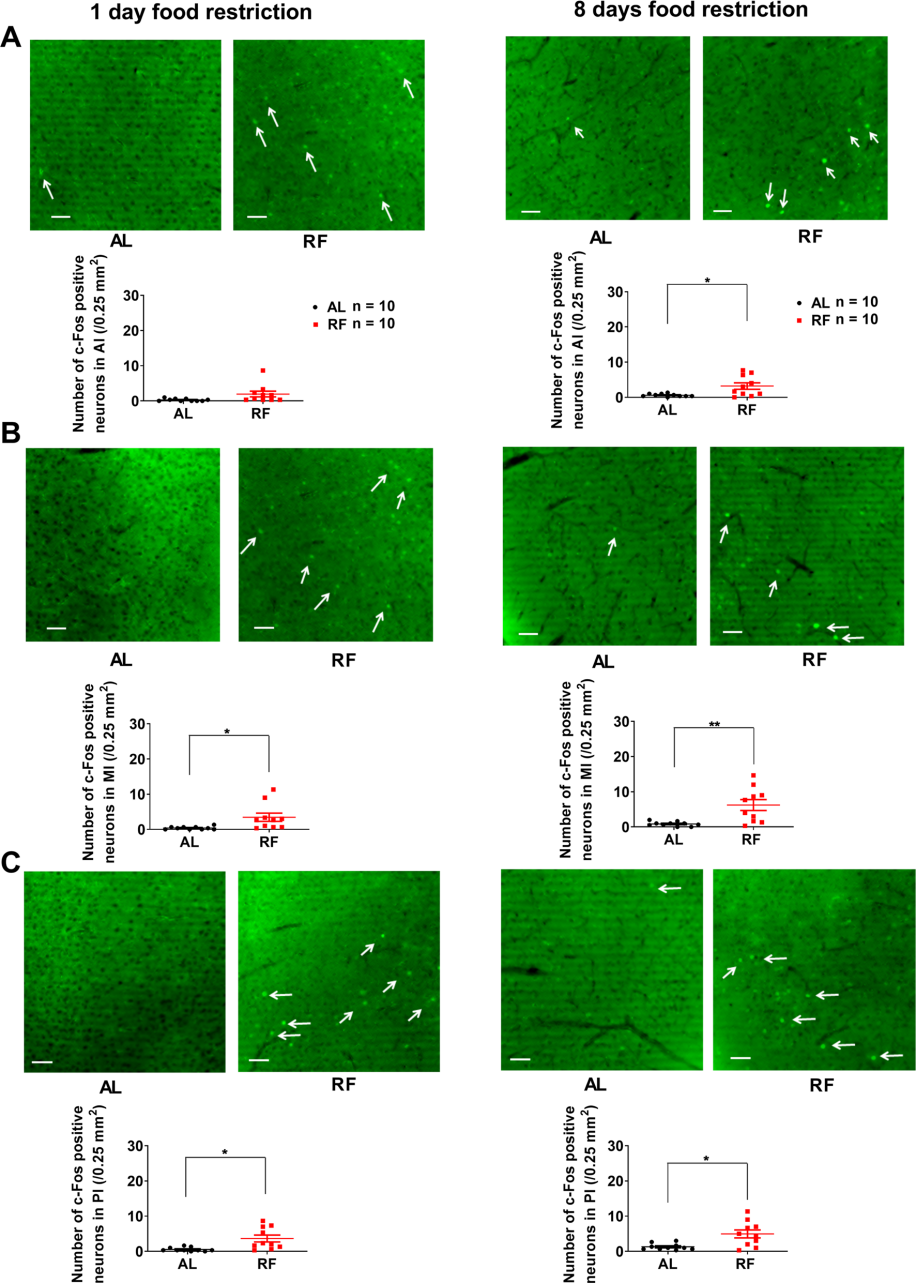
Increase in the number of c-Fos-positive neurons in IC of 1-day or 8 days RF-mice. The mean (± SEM) number of c-Fos positive neurons for ten subjects is shown on the y-axis in the AI (**A**), MI (**B**), and PI (**C**). *P < 0.05; **P < 0.01 difference between AL and RF mice according to unpaired *t*-test with Welch’s correction. Top panels in **A**, **B**, and **C**. Representative sections show c-Fos positive neurons in the AI, MI, and PI of AL and RF mice for 1-day food restriction (Left top) and 8 days of food restriction (Right top), respectively. The scale bar is 50 μm

As shown in Fig. 1B, the appearance of the FAA is gradual. Therefore, we examined if the activation of neurons in the IC of RF-mice was also gradual during the food anticipatory period. We compared two groups on 1st day and 8th day of the feeding restriction protocol (Fig. 5). Compared with AL-mice, the number of c-Fos expressing neurons of 1-day RF-mice was significantly larger in MI (AL: 0.4 ± 0.1/0.25 mm^2^, RF: 3.5 ± 1.2/0.25 mm^2^, *n* = 10, t = 2.578, *P* = 0.0292, unpaired *t*-test with Welch’s correction) and PI (AL: 0.6 ± 0.2/0.25 mm^2^, RF: 3.7 ± 1.0/0.25 mm^2^, *n* = 10, t = 3.111, *P* = 0.0116). There was no significant difference in c-Fos expression between the bilateral MI and PI neurons in 1-day RF-mice. Meanwhile, the number of c-Fos expressing neurons in the left AI of 1-day RF-mice tended to be higher than AL-mice, but no statistical significance (AL: 0.3 ± 0.1/0.25 mm^2^, RF: 2.0 ± 0.8/0.25 mm^2^, *n* = 10, t = 2.030, *P* = 0.0729) (Fig. 5A–C the left). On the other hand, the number of c-Fos expressing neurons in the right AI increased significantly (AL: 0.3 ± 0.1/0.25 mm^2^, RF: 1.2 ± 0.3/0.25 mm^2^, *n* = 10, t = 3.101, *P* = 0.0101). The results indicate that the neurons in the right AI, the bilateral MI, and PI of 1-day RF-mice (fasting for 20 h) are activated during the food anticipatory period.

**Fig. 6:**
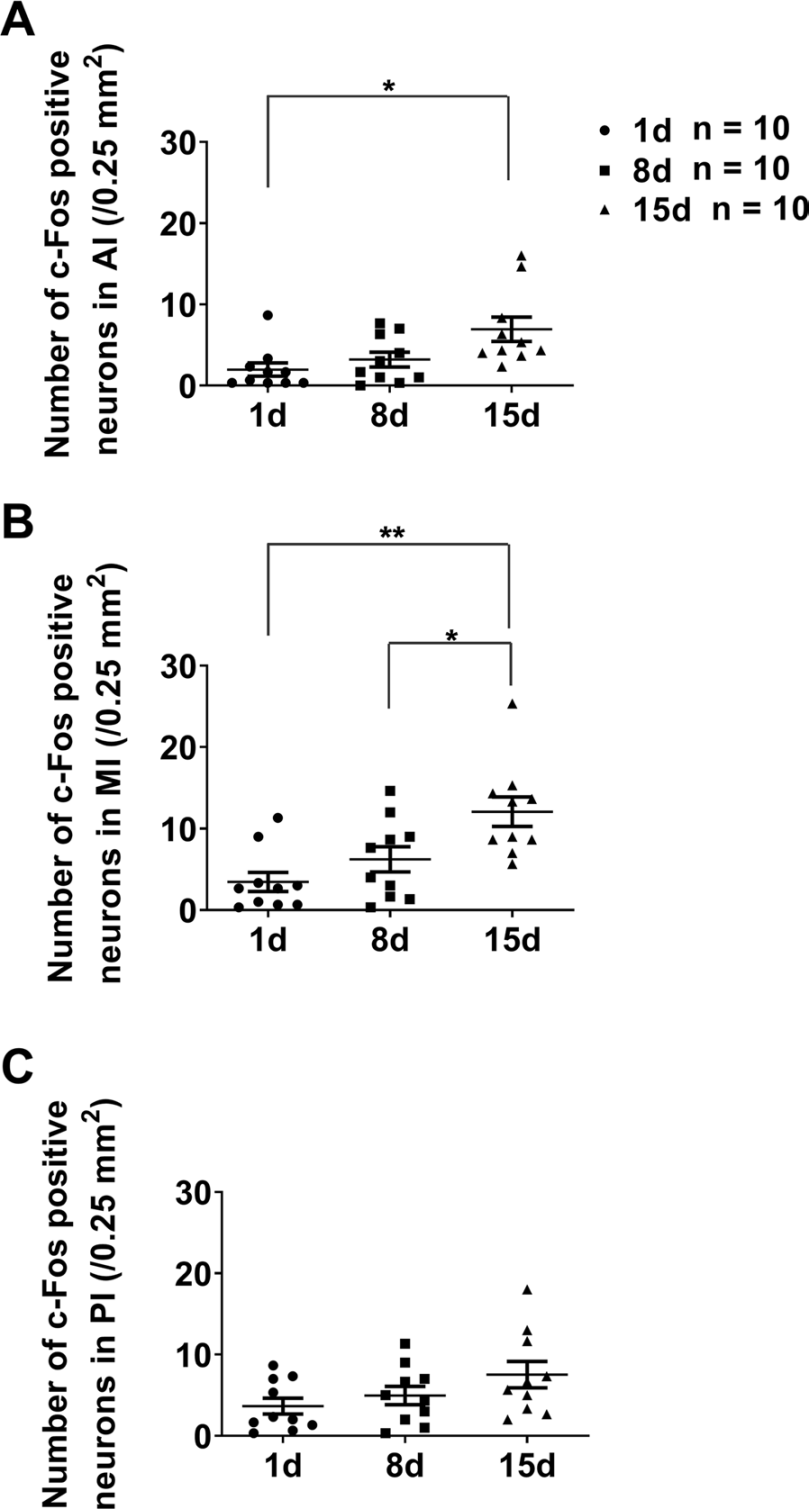
The number of c-Fos-positive neurons in AI/MI/PI of RF mice at 1, 8, and 15 days. The mean (± SEM) number of c-Fos positive neurons for ten subjects is shown on the y-axis in the AI (**A**), MI (**B**), and PI (**C**). Data are reproduction of RF groups shown in **A**-**C** of both Figs. 3 and 5. *P < 0.05; **P < 0.01 difference among the 1 day, 8 days, and 15 days RF mice according to one-way ANOVA with Bonferroni’s multiple comparison test

In 8 days RF-mice, the number of c-Fos positive neurons was significantly larger in AI (AL: 0.6 ± 0.1/0.25 mm^2^, RF: 3.2 ± 0.9/0.25 mm^2^, *n* = 10, t = 2.813, *P* = 0.0195, unpaired *t*-test with Welch’s correction), MI (AL: 0.9 ± 0.2/0.25 mm^2^, RF: 6.2 ± 1.5/0.25 mm^2^, *n* = 10, t = 3.453, *P* = 0.0069) and PI (AL: 1.3 ± 0.3/0.25 mm^2^, RF: 5.0 ± 1.1/0.25 mm^2^, *n* = 10, t = 3.158, *P* = 0.0100) (Fig. 5A–C the right). There was no significant difference in c-Fos expression between the bilateral IC neurons of 8 days RF-mice. The results indicate that the neurons in the bilateral AI, MI and PI are also activated at the 8th day of food restriction during FAA. We then compared the number of c-Fos positive neurons in three-time points to examine the development of c-Fos expression pattern. Among 1 day or 8 days or 15 days RF-mice, the number of c-Fos positive neurons in AI, MI, and PI during the food anticipatory period was the smallest in 1-day RF-mice, followed by 8 days RF-mice, and the largest in 15 days RF-mice (Fig. 6A–C). The results indicate that activation of the neurons in AI, MI, and PI of 1-day RF-mice during the food anticipatory period was at the beginning of the activation development. Compared with the 15 days RF-mice, the number of c-Fos positive neurons in AI of the 8 days RF-mice during FAA was slightly smaller, with no significant difference; but that of the 1-day RF-mice was significantly smaller (*P* = 0.0108, one-way ANOVA; *P* = 0.0119, Bonferroni’s test, Fig. 6A). The results indicate that activation of the neurons in AI of the 8 days RF-mice during FAA was still in the middle of the development of activation. The number of c-Fos positive neurons in the MI was significantly higher in the 15 days RF-mice compared to the 8 days RF-mice and the 1-day RF-mice during FAA, respectively (*P* = 0.0016, one-way ANOVA; *P* = 0.0352 between 15 days RF-mice and 8 days RF-mice, *P* = 0.0014 between 15 days RF-mice and 1-day RF-mice, respectively, Bonferroni’s test, Fig. 6B). The results indicate that activation of the neurons in MI of the 8 days RF-mice during FAA was still in the middle of development of activation. The number of c-Fos positive neurons in PI of the 8 days RF-mice and the 1-day RF-mice during FAA was slightly lower than in the 15 days RF-mice, but there was no significant difference (*P* = 0.1119, one-way ANOVA, Fig. 6C). The results indicate that activation of the neurons in PI of the RF-mice during food anticipatory period didn't significantly increase during our food restriction schedule.

### How does activation of neurons, including orexin neurons in the bilateral LH develop during food restriction protocol?

**Fig. 7:**
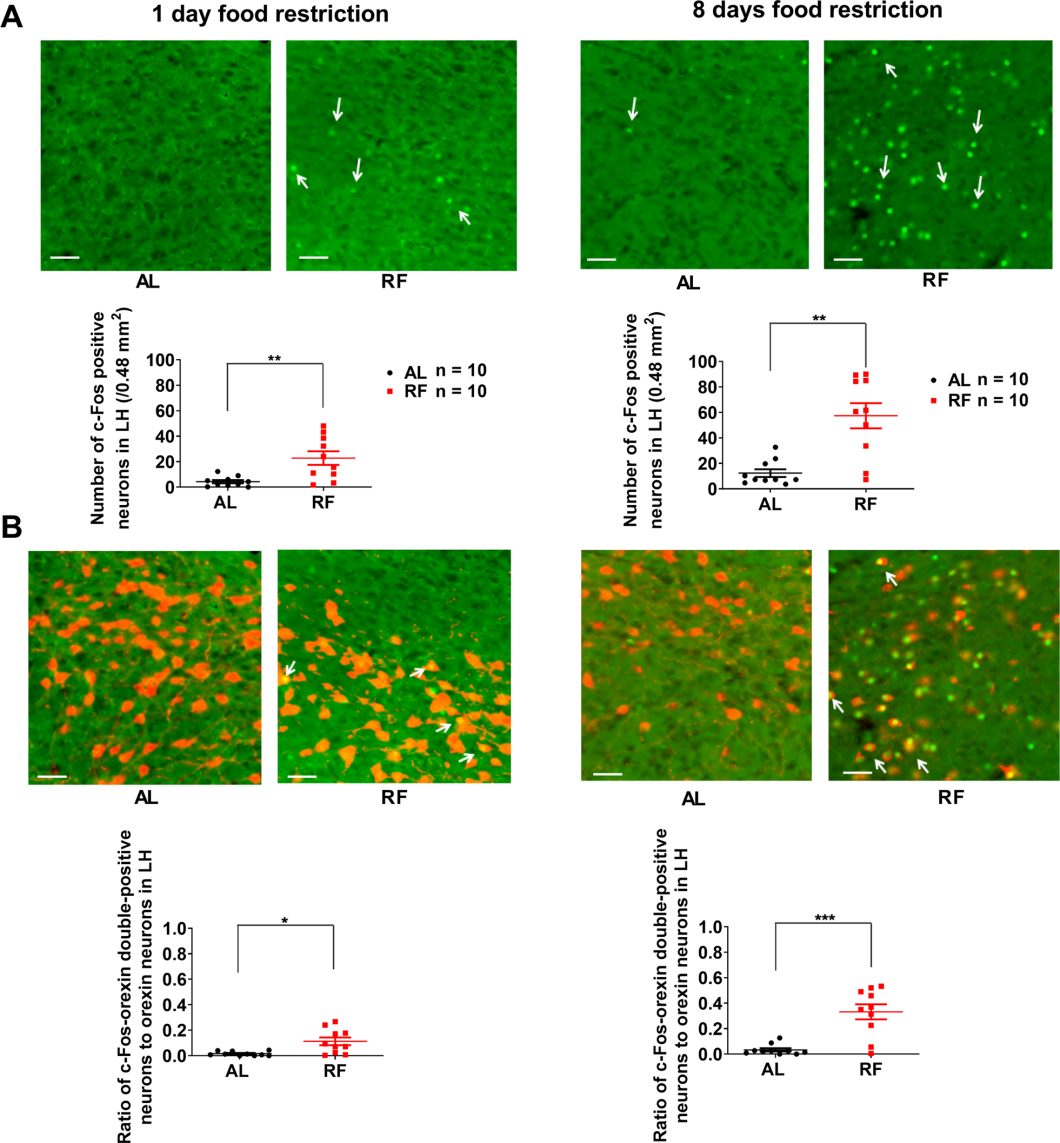
The number of c-Fos-positive neurons and c-Fos-positive orexin-neurons in LH of 1-day or 8-day RF-mice. **A** and **B**, Top. Representative sections show c-Fos positive neurons (**A**) and c-Fos positive orexin neurons (**B**) in the LH of AL mice and RF mice for 1-day food restriction (Left top) and 8 days of food restriction (Right top), respectively. The scale bar is 50 μm. The mean (± SEM) of c-Fos positive neurons (**A**, Bottom) and the ratio of c-Fos-orexin double-positive neurons to orexin neurons (**B**, Bottom) for ten subjects are shown on the y-axis. *P < 0.05; **P < 0.01; ***P < 0.001 difference between AL mice and RF mice according to unpaired *t*-test with Welch’s correction

To research the possible changes in the activation of LH neurons during the food anticipatory period, we compared two groups on 1st day and 8th day of the feeding restriction protocol. On 1st day, the number of c-Fos positive neurons in LH of RF-mice was significantly larger than AL-mice (AL: 4.3 ± 1.3/0.48 mm^2^, RF: 22.8 ± 5.3/0.48 mm^2^, *n* = 10, t = 3.376, *P* = 0.0071, unpaired *t*-test with Welch’s correction) and the proportion of c-Fos positive neurons in orexin neuron was also larger (AL: 0.016 ± 0.005, RF: 0.113 ± 0.030, *n* = 10, t = 3.147, *P* = 0.0109) (Fig. 7A, B the left). There was no significant difference in c-Fos expression between the bilateral LH neurons and orexin neurons of 1-day RF-mice. The results indicate that the neurons, including orexin neurons in the bilateral LH of the 1-day RF-mice are activated during the food anticipatory period.

**Fig. 8:**
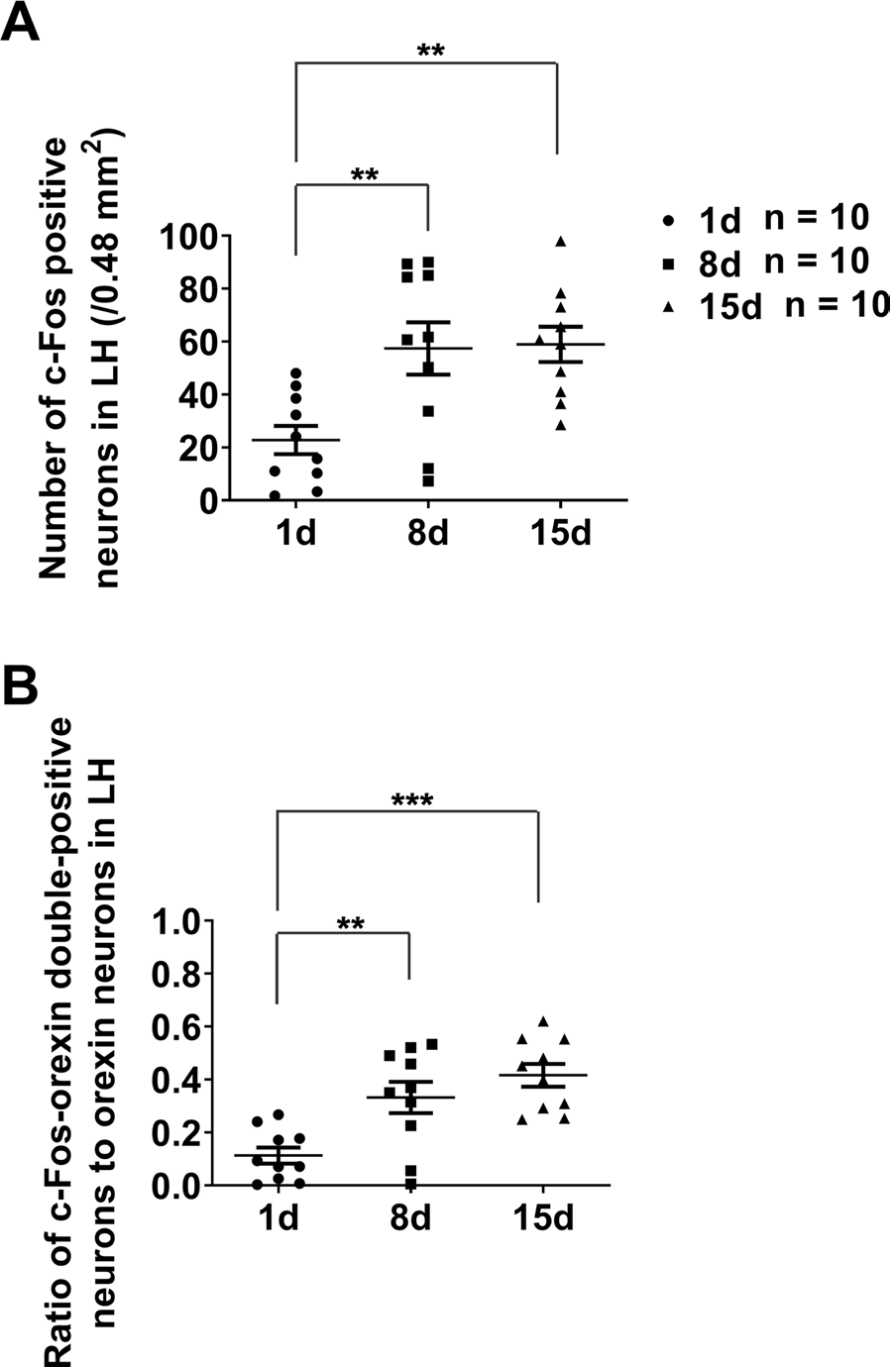
Comparison of the number of c-Fos-positive-neurons and ratio of c-Fos-orexin-double-positive-neurons/orexin-neurons in LH of 1-day/8 days/15 days RF-mice. The mean (± SEM) of c-Fos positive neurons (**A**) and the ratio of c-Fos-orexin double-positive neurons to orexin neurons (**B**) for ten subjects are shown on the y-axis. Data are reproduction of RF groups shown in **A**–**B** of both Fig. 4 and Fig. 7. **P < 0.01; ***P < 0.001 difference among the LH of the 1, 8, and 15 days RF mice according to one-way ANOVA with Bonferroni’s multiple comparison test

On the 8th day, the number of c-Fos positive neurons (AL:12.3 ± 3.1/0.48 mm^2^, RF: 57.4 ± 9.9/0.48 mm^2^, *n* = 10, t = 4.362, *P* = 0.0014) and proportion of c-Fos positive orexin neurons (AL: 0.033 ± 0.013, RF: 0.332 ± 0.059, *n* = 10, t = 4.948, *P* = 0.0006, unpaired *t*-test with Welch’s correction) in RF-mice were significantly larger as compared with AL-mice (Fig. 7A, B the right). There was no significant difference in c-Fos expression between the bilateral LH neurons and orexin neurons of 8 days RF-mice. The results indicate that the neurons in the bilateral LH including orexin neurons of the 8 days RF-mice are activated during FAA. Compared with the 15 days RF-mice, the 8 days RF-mice showed slightly smaller c-Fos. Still, the difference did not reach a significant difference (*P* > 0.05, Bonferroni’s test, Fig. 8). The results indicate that activation on day 8 had almost already reached the plateau. Among 1 day, 8 days, or 15 days RF-mice, the number of c-Fos positive neurons in LH in 1-day RF-mice was the smallest. There was a significant difference compared with 8 days or 15 days RF-mice (*P* < 0.01, *P* < 0.001, one-way ANOVA with Bonferroni’s test, Fig. 8). The results indicate that activation of the LH neurons, including orexin neurons started from day 1 of RF and reached the plateau around day 8 of RF.

### The relationship between the number of c-Fos positive IC neurons and orexin neurons in the production of FAA

**Fig. 9:**
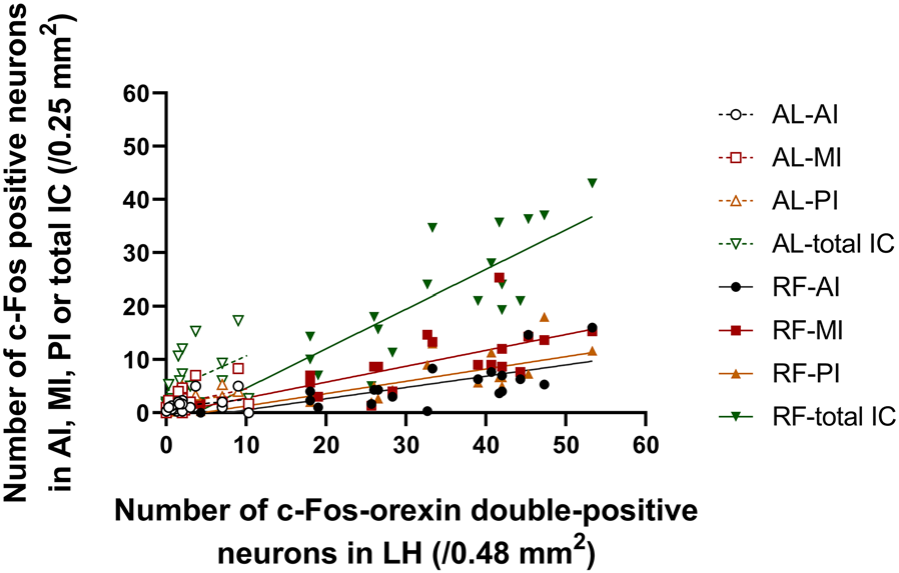
Correlation of c-Fos expression between neurons in IC and orexin neurons during FAA. The number of c-Fos-orexin double-positive neurons in LH of the 8 days RF mice (*n* = 10) and their control AL mice (*n* = 10), and 15 days RF mice (*n* = 10) and their control AL mice (*n* = 10) are shown on the x-axis, respectively. The number of c-Fos positive neurons in AI, MI and PI in IC of the 8 days RF mice (*n* = 10) and their control AL mice (*n* = 10), and 15 days RF mice (*n* = 10) and their control AL mice (*n* = 10) are shown on the y-axis, respectively. Data of total IC are the sum of AI, MI and PI. RF-mice showed a significant positive correlation between the number of neurons activated in AI or MI or PI or total IC and the number of orexin neurons activated in LH (*P* < 0.001), but AL-mice did not (*P* > 0.05) according to Pearson's correlation coefficient

To investigate the interaction between IC neurons and LH orexin neurons in FAA, we compared their activation during the food anticipatory period using FAA-established day 8 and 15 data. RF-mice showed a significant positive correlation between the number of neurons activated in AI or MI or PI or total IC and the number of orexin neurons activated in LH (AI, r = 0.7088, *P* = 0.0005; MI, r = 0.7117, *P* = 0.0004; PI, r = 0.7348, *P* = 0.0002; total IC, r = 0.8582, *P* < 0.0001, Pearson's correlation coefficient), but AL-mice did not (AI, r = 0.3322, *P* = 0.1525; MI, r = 0.4398, *P* = 0.0523; PI, r = 0.3606, *P* = 0.1183; total IC, r = 0.4251, *P* = 0.0617) (Fig. 9). These significant positive correlations were similar in the bilateral hemisphere of RF-mice. The results indicate an interaction between the activation of neurons in the IC (including AI, MI, PI) and the activation of orexin neurons of RF-mice during the FAA.

### Trends in changes in locomotor activity, food intake, and body weight of mice in the short version of the food restriction protocol

**Fig. 10:**
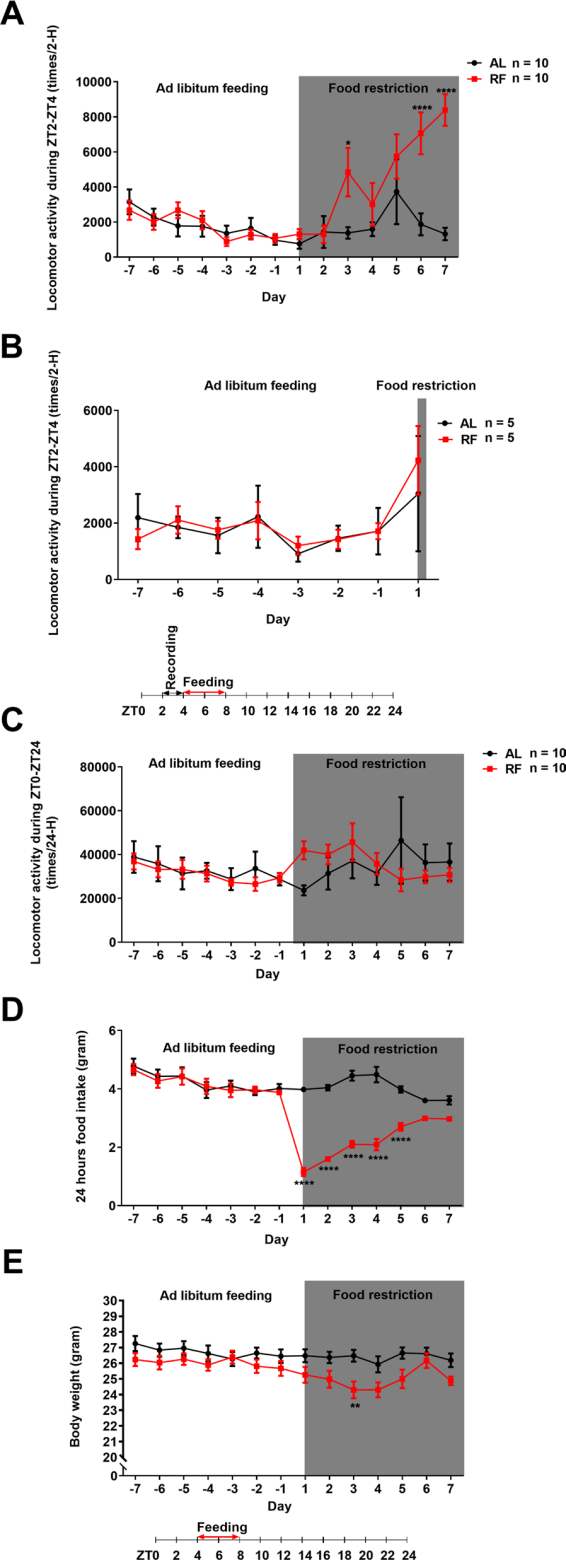
Daily locomotor activity, food intake and body weight of mice during1 or 8-days restricted feeding. **A** and **B** Increased food anticipation activity (locomotor activity during ZT2-ZT4) in response to the restricted feeding in 8 days (**A**, *n* = 10) or 1 day (**B**, *n* = 10 during ad libitum feeding, *n* = 5 during food restriction) RF mice. **C** A similar daily total locomotor activity was observed in the 8 days RF mice to that of the AL mice. **D** Daily food intake fluctuation and (**E**) changes in daily body weight of the 8 days RF mice. The mean (± SEM) of daily food intake (**D**) or daily body weight (**E**) for ten subjects is shown on the y-axis. The x-axis represents experimental days. *P < 0.05 (A); ****P < 0.0001 (**A** and **D**); **P < 0.01 (**E**) difference between AL mice and RF mice according to repeated measures two-way ANOVA with Bonferroni’s multiple comparison test. According to repeated measures two-way ANOVA, there was no significant difference in locomotor activity during ZT2-ZT4 (**B**) or ZT0-ZT24 (**C**) between AL mice and RF mice

To examine whether the trends were similar to those of the 15 days RF-mice, we investigated the effects of the daily scheduled RF for 8 days or 1 day on the locomotor activity, food intake, and body weight of mice. Compared with AL-mice, the locomotor activity during ZT2-ZT4 of 8 days RF-mice increased significantly (feeding-effect: F(1, 18) = 8.066, *P* = 0.0109; day-effect: F(13, 234) = 7.616, *P* < 0.0001; interaction: F(13, 234) = 5.537, *P* < 0.0001, repeated measures two-way ANOVA, Fig. 10A). It increased significantly after 2 days of RF (*P* = 0.0187, Bonferroni’s test), then remained at a higher level than that of AL-mice during RF (*P* < 0.0001), which was similar to the results observed in the 15 days RF-mice, although a significant increase in the locomotor activity of 15 days RF-mice began after 3 days of RF. However, the locomotor activity during ZT2-ZT4 of 1-day RF-mice was no significant difference (feeding-effect: F(1, 18) = 0.04694, *P* = 0.8309; day-effect: F(7, 116) = 3.935, *P* = 0.0007; interaction: F(7, 116) = 0.5740, *P* = 0.7758, repeated measures two-way ANOVA; *P* > 0.05, Bonferroni's test, Fig. 10B). The result suggests that the 1 day feeding restriction program, i.e., fasting for 20 h, in this study cannot affect the locomotor activity during ZT2-ZT4 in mice, and cannot form FAA, which is similar to that observed in the 15 days or 8 days RF-mice. The results also demonstrated that the formation of FAA in the 15 days or 8 days RF-mice was not due to the 20-h fasting just before the brain sampling but was due and specific to the food restriction protocol. The fluctuation of the FAA was also observed in 8 days RF-mice. In addition, the 8 days of food restriction between ZT4 and ZT8 did not affect the 24-h total ambulatory activity of mice (feeding-effect: F(1, 18) = 0.0007720, *P* = 0.9781; day-effect: F(13, 234) = 0.9675, *P* = 0.4842; interaction: F(13, 234) = 1.399, *P* = 0.1607, repeated measures two-way ANOVA, Fig. 10C), which is similar to the results observed in the 15 days RF-mice.

The daily food intake of the 8 days RF-mice decreased significantly on the first day of food restriction (feeding-effect: F(1, 18) = 36.40, *P* < 0.0001; day-effect: F(13, 234) = 34.76, *P* < 0.0001; interaction: F(13, 234) = 26.56, *P* < 0.0001, repeated measures two-way ANOVA; *P* < 0.0001, Bonferroni’s test), then recovered gradually with the number of days and reached a similar level to that of AL-mice after 5 days of feeding restriction (*P* > 0.05, Fig. 10D), which are similar to those in the 15 days RF-mice.

The daily body weight of the 8 days RF-mice decreased significantly by 6.7% on the first three days of feeding restriction (feeding-effect: F(1, 18) = 4.140, *P* = 0.0569; day-effect: F(13, 234) = 6.580, *P* < 0.0001; interaction: F(13, 234) = 2.365, *P* = 0.0055, repeated measures two-way ANOVA; *P* = 0.0083, Bonferroni’s test), then recovered gradually with the number of the days (up to 0.5%) (*P* > 0.05 (day 4), Fig. 10E). Although the maximum daily body weight loss of 8 days RF-mice was on the third day of feeding restriction, and that of 15 days RF-mice was on the first day of feeding restriction, the changing trend of daily body weight of mice in these two groups during the RF was similar. The RF between ZT4 and ZT8 caused a decrease in the body weight only in the early stage of the RF.

## Discussion

To investigate the functional role of the insular cortex and lateral hypothalamus in FAA production, we examined the neuronal activation of these areas during the food anticipatory period. This study demonstrates that the development of FAA is a gradual process and requires periodic, predictable feeding restriction stimulation. In addition, an increase of c-Fos-positive neurons in the IC and LH during the food anticipation period was gradual from the 1st day to the 15th day of restricted feeding. There was a positive correlation between the activation of neurons in IC and orexin neurons in LH during the food anticipation period. However, the increase in c-Fos-positive neurons of these brain regions changed differently during the food restriction protocol. These results suggest that the insular and lateral hypothalamic neurons, including orexin neurons, are active during FAA, and the IC and LH are differently involved in the neural network for FAA production.

We examined the formation and development of FAA in mice during food restriction protocol. Previous studies have shown that food-related cues are powerful time signals of physiological and behavioral systems [12, 38, 39]. The scheduled daily access to food causes FAA. The FAA is characterized by behavioral arousal and activation, increased locomotion, increased proximity to a feeder, and food seeking [38, 39]. The current study showed that 1-time food restriction, i.e., fasting for 20 h, did not affect locomotor activity during the pre-feeding period in mice and did not result in FAA formation. However, periodic RF applied at least two to three times significantly increased locomotor activity during the pre-feeding period. This indicates that the FAA is not a result of the 20-h fast but rather from the feeding restriction protocol used in this study. It was also observed that the RF did not affect the movement capability but could cause significant alterations in daily locomotor activity patterns (an enhancement of locomotor activity during the light period), similar to the previous study [12].

In addition, this study shows that the neurons in three insular subregions are activated during FAA, which is the first report. Therefore, the IC may contribute to the network of brain regions involved in FAA. The mechanism of FAA production caused by daily RF at a fixed time is still unclear. Previous studies suggest that the food entrainment oscillator (FEO) is located outside the suprachiasmatic nucleus (SCN) because the damage to the SCN cannot eliminate FAA [42]. FEO regulates behavior, tissue, cellular, and molecular processes in response to food intake patterns [38]. FEO may have a distributed organization (multiple brain areas including the arcuate nucleus and LH, et al.) and not rely on a single nucleus [12, 35, 36], and damage to one brain area does not eliminate all manifestations of food entrainment [43]. There is a report that food expectation cues significantly regulate the activity of the IC in mice which is necessary for food cues to induce behavioral responses [44]. However, there are few reports on whether the IC participates in FAA [19]. The previous study showed that the inactivation of male Wistar rats' bilateral anterior agranular IC by electrolysis or ibotenic acid lesions significantly increased the FAA [19]. The present study observed a significant increase in c-Fos expression of the bilateral AI neurons of mice during FAA, suggesting the AI neurons are activated during the period. Functionally distinct regions inside the AI might contribute to this disparity between the two studies [21, 45]. The present study observed a few more c-Fos positive neurons in the III and V layers of the AI (data not shown). Manipulating the activity of neurons in the sub-area with Optogenetics or Chemogenetics during FAA might help to answer the question.

We also observed that the developmental process of c-Fos expression differed slightly between the three insular subregions. The number of c-Fos positive neurons in AI gradually increased from 1 to 15 days of food restriction, with 15 days of RF-mice having significantly more than 1-day RF-mice. The number of c-Fos positive neurons in MI also gradually increased from 1 to 15 days of RF. The exposure to RF for 15 days was especially remarkable. However, c-Fos positive neurons in PI only showed a tendency of increasing gradually from 1 to 15 days of RF. There are significantly more c-Fos positive neurons on the 1-day RF-mice in PI compared with AL-mice, the activation of these neurons during the food anticipatory period may reach the peak of activation at the early phase of food restriction. These data suggest that the MI neurons may be more sensitive to repeated RF protocol than the AI and PI. It is well known that there are functional differences between these three areas [20, 22–25]. However, the exact mechanisms that induce different changes in the three subregions of the IC need to be further studied.

Although in the present study, 1-day food restriction caused an increase in c-Fos expression of the bilateral AI neurons, it was only significant on the right side, suggesting the right AI neurons can be more sensitive to the hunger signal. There have been some reports about the asymmetric activation pattern of the IC [22, 46–48]. For example, human functional magnetic resonance imaging showed that interoceptive attention induced similar significant activation in the bilateral IC, with the highest degree of activation in the middle short gyrus, followed by the anterior and posterior short gyri. However, the interoceptive accuracy induced significant activation of the right dorsal AI [48]. The present study and previous studies have shown that there may be some functional differences between the bilateral AI.

This study also shows that the LH neurons, including orexin neurons, are gradually activated during FAA by the food restriction protocol. Orexin neurons in LH are associated with arousal [49–52], and are part of the “approach-exploratory” system that regulates muscle tone and motor behavior [53, 54]. It has been reported that mice with orexin neurons ablated had a severe defect in showing expected food-anticipatory increases in locomotor activity [55, 56] and wakefulness [56] under RF conditions. Orexin neuron is necessary for the strong expression of locomotor activity in anticipation of feeding [33]. Orexin neurons in LH are activated during food anticipation and exhibit self-sustained oscillations driven by food-entrainment [37]. The current data would also support previous findings that neurons including orexin neurons in LH participate in FAA [12, 33, 37, 55, 56]. The number of c-Fos-positive orexin neurons increased with the days of RF, reached a maximum, and remained stable after eight days of RF, which suggests the activation of orexin neurons in the LH during FAA is maintained at a high level after 8 days of RF protocol. The data that 1-day RF already activates the orexin neurons suggests their involvement in hunger-induced foraging motivation and behavior [31]. Interestingly, the present study also found that non-orexin-neurons in the LH were activated during FAA with the same activation tendency as orexin neurons. As a result, more research is needed to determine how different neurons in the LH contribute to FAA.

The mechanism of FAA production still needs to be clarified. We hypothesize the possible mechanisms of FAA production caused by daily scheduled RF. It has been reported that FAA is regarded as the output of FEO [12, 38]. The present study suggests that neurons in IC and LH, including orexin neurons, may be a part of FEO. In our research, the FAA became obvious from day 3 to day 4 of food restriction and remained higher until the 15th day. On the other hand, the increase in the number of c-Fos positive neurons in IC and LH, including orexin neurons, during the food anticipatory period began from day 1 of food restriction. It peaked at a different phase of the food restriction protocol. According to the beginning time, the activation of IC and LH neurons is faster than the formation of FAA, suggesting that the number of activated neurons in IC and LH is insufficient to cause FAA formation on the first day of RF. As the number of days of RF increases, the number of activated neurons in IC (especially in AI and MI subregions) and LH gradually increases and might be enough to cause the formation of FAA on the third or the fourth day of RF. How the increased number of activated neurons in the brain areas causes the formation of FAA needs further research.

Interestingly, c-Fos expression of the IC neurons during the food anticipatory period peaked on the 15th day of food restriction. On the other hand, c-Fos expression of the LH neurons, including orexin neurons, peaked on the 8th day of food restriction. That is, the development process of the activation in the IC neurons was slower than that in the LH neurons, including orexin neurons. The nerve fibers of orexin neurons in LH project to IC [57, 58]. The increased orexin transmission in the IC of aged rats can enhance feeding behavior by significantly reducing the feeding latency [59]. According to the activation pattern of the IC neurons and the LH neurons during FAA in the present study, we speculate that the LH neurons, including orexin neurons, could receive food entrainment information from receptors and brain regions that sense internal information (such as hunger or weight loss or Zeitgeber, etc.), awaken animals during food anticipatory period. Then this information might be transmitted to the IC by the orexinergic nerve fibers projected from the LH [57, 58] and exciting neurons in the IC [59] to be involved in FAA. In addition, there was a positive correlation between the number of neurons activated in the IC and orexin neurons during FAA, suggesting there is an interaction between the activation of neurons in the IC (including AI, MI, PI) and orexin neurons during the food anticipation period. Another possibility is that the activation of the LH and IC developed independently and supported their different functional roles in FAA production [12, 19, 33]. Therefore, the exact mechanism of the involvement of the IC and LH in FAA production and the relationship between them during FAA needs to be further studied.

Before the experiment, we speculated that RF mice might reduce their daily food intake and body weight due to RF compared to AL mice. However, in the present study, even under the condition of time-limited access to food, the daily food intake of the RF-group increased to the same level as the AL-group, and the daily body weight of the RF-group increased to a level higher than that of the AL-group in the late stages of the protocol. Furthermore, the present study showed activation of the neurons in the IC and LH, including orexin neurons, during FAA. Therefore, we hypothesize the following mechanism. The activation of the IC and LH during the last stage of the food restriction protocol may compensate for the reduction of eating amount and increase of energy consumption during the early phase of RF. In particular, the information about reducing food intake and increasing energy use was sent to excite orexin neurons in the LH [31], which then sent the information to the IC [57, 58] and excited neurons in the IC [59]. This might lead to the recovery of food intake [14, 15, 28, 31, 60] during the scheduled feeding period and an increase in the daily body weight of mice in the late stage of the RF.

### Limitations of the experiments

Most previous studies on FAA have used male rodents [12, 35–37, 39, 61], and a few studies reported no significant differences in FAA between male and female mice [33]. However, it has also been reported that male mice show significantly more FAA than female mice [62]. Although this study only used male mice, it would be valuable to compare FAA development between male and female mice.

In the study of the developmental process of c-Fos expression in IC and LH neurons during the food anticipatory period, we examined the changes in c-Fos expression in these brain areas on the 1st, 8th, and 15th day of RF (Fig. 6; Fig. 8). The findings of this study provide insights into the developmental dynamics of c-Fos expression in neurons located in the IC and LH regions during the food anticipation period. However, future experiments with more time points may yield more comprehensive and conclusive results regarding the developmental process of c-Fos expression in neurons within this specific brain area during the food anticipation period.

## Conclusion

In summary, this study demonstrates that the bilateral insular and lateral hypothalamic neurons, including orexin neurons, are active during FAA. The temporal patterns of neuronal activation in several subregions of the IC are different, and those of neuronal activation between the IC and LH are also different, suggesting that the IC and LH are differently involved in the neural network for FAA production.

## Data Availability

Summary statistics are available in the article. In addition, the raw data supporting the findings in this study are available from the corresponding author on reasonable request.
